# Disease-preventive behaviors and subjective well-being in the COVID-19 pandemic

**DOI:** 10.1186/s40359-023-01316-x

**Published:** 2023-09-25

**Authors:** Matthew Tokson, Hadley Rahrig, Jeffrey D. Green

**Affiliations:** 1 S.J. Quinney College of Law, University of Utah, Salt Lake City, UT USA; 2 Psychology Department, University of Wisconsin-Madison, Madison, WI USA; 3 Psychology Department, Virginia Commonwealth University, Richmond, VA USA

**Keywords:** Well-being, Life satisfaction, Meaning, Covid, Prosociality, Anxiety

## Abstract

**Background:**

Safety precautions and activity restrictions were common in the early, pre-vaccine phases of the COVID-19 pandemic. We hypothesized that higher levels of participation in potentially risky social and other activities would be associated with greater life satisfaction and perceived meaning in life. At the same time, prosocial COVID-preventive activities such as mask wearing should enhance life satisfaction.

**Method:**

We assessed the impact of COVID-preventive behaviors on psychological well-being in October 2020. A nationally representative sample of U.S. adults (*n* = 831) completed a demographic questionnaire, a COVID-related behaviors questionnaire, a Cantril’s Ladder item, and the Multidimensional Existential Meaning Scale. Two hierarchical linear models were used to examine the potential impact of COVID-preventive behaviors on life satisfaction and meaning in life while accounting for the influence of demographic factors.

**Results:**

The study revealed significant positive relationships between COVID-preventive behaviors and subjective well-being. Wearing a mask was significantly associated with higher life satisfaction, while maintaining social distancing of six feet and avoiding large groups were significantly associated with higher perceived meaning in life. Social activities including dining at restaurants and visiting friends and family were also significantly associated with higher life satisfaction and meaning in life, respectively.

**Conclusion:**

The study’s findings support the conclusion that disease prevention measures such as social distancing and mask wearing do not reduce, and may enhance, subjective well-being during a pandemic. Utilizing the unique context of the COVID-19 pandemic to examine relationships between behavior and subjective well-being, the study also indicates that shallow or medium-depth social activities are likely to be more central to life satisfaction, whereas narrower, deeper social interactions with friends and family are more important to perceived meaning in life.

**Supplementary Information:**

The online version contains supplementary material available at 10.1186/s40359-023-01316-x.

## Background

The early, pre-vaccine period of the COVID-19 pandemic gave rise to several atypical behavioral responses, including social distancing, mask wearing, and the avoidance of indoor social activities. These behaviors likely slowed the spread of COVID and greatly reduced the loss of life. Yet these actions and restrictions may come with substantial psychological costs. Socialization, work, and non-verbal communication via facial expression have been associated with psychological well-being, and restrictions on these behaviors may negatively impact psychological health and disincentivize engagement in public health behaviors (E.g., [1–3]). The present study explores this potential trade-off by examining the impact of COVID-avoidant behaviors on psychological well-being.

In the fall of 2020, the “third wave” of the COVID-19 pandemic was beginning to rise. A surge of cases began in the Upper Midwest in September 2020. It soon spread to the entire nation, and continued to rise rapidly throughout the fall and early winter, eventually becoming the deadliest wave of the pandemic to date. It lasted for months until acquired immunity, warmer weather, and the availability of vaccines led the wave to finally recede in the early spring of 2021 [4]. The present study was conducted during the early portion of this period, in October of 2020.

Responses to the COVID-19 pandemic varied widely across different nations, with some countries imposing and enforcing aggressive behavioral restrictions and others imposing moderate restrictions on businesses and some legal restrictions on personal behavior. The United States adopted a primarily voluntary approach to addressing COVID, with relatively few government restrictions on individual behavior and very little enforcement of those restrictions [5].

Correspondingly, COVID-preventive behaviors such as mask wearing and social distancing were largely undertaken voluntarily in the United States, as well as many other western countries. These behaviors appear to have been motivated by the circumstances of the pandemic, and especially by rising infection rates, rather than by legal requirements or government policies [6, 7]. Accordingly, self-reported mask wearing was associated with significantly reduced COVID transmission in a study of several countries, but mask mandates were not [8].

Throughout the pandemic, social distancing, mask wearing, and other measures taken by individuals were somewhat effective in slowing the spread of COVID, according to existing studies and assessments [7–9]. However, these measures may be associated with some degree of psychological harm. Socializing with others, close contact and touch, going to work, exercising at a gym, and caring for family members can all contribute to psychological well-being, and restrictions on these behaviors may reduce psychological health [2, 3, 10–12]. Mask wearing, notwithstanding its substantial health benefits in a pandemic, may be physically unpleasant or may engender feelings of restriction or anxiety [13, 14]. Mask wearing also proved politically controversial, as sporadic anti-mask protests arose across the country in 2020 and some mask skeptics engaged in violent noncompliance with indoor mask mandates [15, 16]. Some researchers have theorized that masks may reduce well-being by impairing social interactions and reducing empathy and comprehension, or by increasing pandemic-related stress or depression [13, 17].

On the other hand, people are generally psychologically resilient, and even substantial changes to their daily routine may not, on average, significantly decrease their life satisfaction [18]. Although the precise mechanisms of this resilience are unknown, complete or partial psychological adaptation to adverse circumstances has been observed in numerous settings [19–22]. Consistent with this phenomenon, overall levels of life satisfaction remained stable during most of 2020, dropping in the first month or so of the pandemic but then largely returning to the same level as in previous years (e.g., [1, 18, 23]). Yet it is unknown whether COVID-avoidant behaviors raised or lowered life satisfaction for individuals. The unusual circumstances of the pandemic and widespread COVID-related behavioral restrictions provide a unique context in which to study the effects of various behaviors and responses on well-being.

### Well-being, behavior, and the pandemic

Subjective well-being (SWB) refers to people’s cognitive and affective evaluations of their lives [24]. SWB can take several forms, from positive affect and emotion, to satisfaction with one’s life, to engagement in interesting activities, to experiencing life as meaningful. The present study examines two central aspects of SWB, life satisfaction and meaning in life, each of which has been widely studied (e.g., [10, 22, 25–27]).

Life satisfaction refers to the subjective cognitive assessment of a person’s life as a whole [28]. Judgments of satisfaction generally involve a comparison of one’s circumstances with one’s internally chosen criteria for a good life [29].

Perceived meaning in life refers to a cognitive and emotional assessment of whether one’s life has purpose and value [28]. It examines the subjective experiences of human beings and asks what makes them experience meaning in their lives [30]. In recent years, a consensus has formed around a tripartite definition of perceived meaning in life. Under this definition, lives are experienced as meaningful when they are felt 1) to have purpose, 2) to have significance, and 3) to be coherent [26, 30–32]. Purpose refers to having goals, direction, or a mission in life that extends into the future. Significance refers to feelings of existential mattering, feelings of mattering in the social world, and a sense of generativity, i.e., making contributions to others that extend beyond one’s personal existence [30, 31]. Coherence involves making sense of one’s experiences in life, based on an integrative understanding of one’s self and the world [33, 34]. Together, these concepts reflect the larger concept of a meaningful life, a life that makes sense, has purpose and larger goals, and matters in the larger social or existential sense. The present study examines life satisfaction and meaning in life in the context of the COVID-19 pandemic.

To date, there has been little research on COVID-avoidant behaviors and SWB. Newman et al. (2021), found that higher meaning in life scores on a single-item survey question were associated with more engagement in a broad set of preventative health behaviors and less engagement in risky health behaviors in the earliest months of the pandemic [35]. That study did not examine relationships between meaning and individual behaviors. The study did find that preventive health behaviors were positively associated with negative affect, likely because cancelling social plans and avoiding others increases negative feelings. Risky health behaviors, like socializing with others outside the home, were negatively related to negative affect, presumably because they enhance emotional well-being [35]. Baños et al. (2023), found that meaning in life decreased over time in a longitudinal study conducted in Spain during a strict lockdown period [36]. Meaning stopped decreasing and plateaued during the subsequent twenty days, as restrictions on movement were gradually relaxed.

A handful of studies conducted during the COVID pandemic have examined the effects of specific behaviors on well-being, although these studies do not assess COVID-specific behaviors such as mask wearing or social distancing. In a study of 55,204 UK adults conducted in the early weeks of the pandemic, time spent working, volunteering, doing housework, gardening, exercising, reading, engaging in hobbies, and communicating remotely with family and friends were all associated with increased life satisfaction [37]. In a study of Irish adults (*n* = 604) conducted March 2020, spending time outdoors, exercising, gardening, pursuing hobbies, and taking care of children were associated with greater emotional well-being, while time spent home-schooling children was associated with reduced emotional well-being [38]. A study conducted in China in February 2020 found that respondents (*n* = 369) who continued working in an office had higher life satisfaction than those who had stopped working [39].

In non-pandemic contexts, research linking social interactions and personal activities to higher SWB suggests that a decrease in such activities may be associated with reductions in SWB (e.g., [28, 37, 40, 41]). Relatedly, prosocial behaviors have often been associated with increased SWB. Giving money or other forms of assistance to others has been linked to greater happiness and meaning in life in a variety of observational and experimental studies [42–44]. Behaviors such as charitable giving are associated with higher SWB [42–44], and altruistic COVID-preventive activities such as mask-wearing may also benefit well-being.

### The present study

The primary goal of the present study was to examine COVID-avoidant behaviors in the midst of the pandemic and to examine the relationship between these behaviors and the life satisfaction of American adults. A secondary goal of the study was to investigate the relationship between COVID-avoidant behaviors and perceived meaning in life. Understanding how various behaviors interact with these forms of SWB can shed light on the sources and correlates of well-being. By examining the strength of relationships between pandemic-related behaviors and these forms of SWB, we may be able to better understand which aspects of daily life are associated with well-being, and how everyday behaviors are linked to psychological welfare. Based on prior findings indicating that activities, especially social-interactive activities, are associated with elevated SWB, we hypothesized that higher levels of participation in activities such as going to work, visiting friends and family, attending church, going to the gym, and going to restaurants would be associated with greater life satisfaction and greater meaning in life. Conversely, we expected that COVID-preventive practices that reduce social contact, such as remaining six feet away from others or avoiding indoor gatherings of ten or more persons, would be negatively associated with life satisfaction (e.g., [41]). The relationship between these activities and meaning in life is more ambiguous, as higher meaning in life has been linked to increased social distancing in the early months of the pandemic [35]. Mask wearing during a pandemic is in part a prosocial, altruistic behavior, which we expected to be associated with enhanced life satisfaction (e.g., [42, 43]). The overall correlation between mask wearing and life satisfaction was uncertain, however, given the inconvenience, anxiety, and social inhibition potentially associated with mask wearing [13, 14, 17]. Based on prior studies of the early pandemic period, we expected that mask wearing behavior would have no significant correlation with meaning in life [35].

## Method

### Participants and procedure

The study participants were a nationally representative sample of 831 United States adults from all regions of the country recruited by Qualtrics. Qualtrics compiles samples from various market and academic research panels. The survey took, on average, approximately 8.5 min to complete, and participants were compensated $4 upon completion. Participants signed an electronic consent form and completed an online survey. Of the 1019 participants originally recruited for the study, participants who failed to answer questions, completed the survey unfeasibly quickly, or failed a bot screening test were screened out and not included in the analysis (*n* = 188). In addition, 60 respondents completed an early version of the survey that did not include the Multidimensional Existential Meaning Scale (MEMS) questions, 5 respondents did not report their age, and 6 respondents did not complete all COVID behavior questions. Missing data were addressed via pairwise deletion.

Sample size selection was guided by similar, prior survey-based study designs [38, 45, 46]. Post-hoc sample size estimation [47], using the conservative parameters of 5% margin of error, 50% response distribution, and a 99% confidence interval, suggested a minimum *N* of 643 was adequately powered to detect modest effect sizes. Accordingly, recruiting 1000 participants is appropriate assuming a retention rate of 70%, to account for low quality responses.

All instruments and procedures were approved by the University of Utah’s Institutional Review Board. Participants were asked a series of questions about demographics and COVID-related behaviors, and they completed the SWB measures described below. Surveys were administered from October 14–27, 2020. Table 1 presents the demographic characteristics of the participants.

**Table 1:** Participant demographics (*n* = 831)

**Categorical Measures**	***n***	**%**
Gender		
Male	400	48.1
Female	428	51.5
Non-Binary/Other	3	.4
Race/Ethnicity		
Asian	49	5.9
Black or Af. Am	100	12.0
Hispanic or Latino	144	17.3
White	498	57.9
Other	40	4.8
Education		
High school degree (or less)	205	24.7
Associate’s degree or some college	205	24.7
College degree	199	23.9
Graduate studies or graduate degree	222	26.7
Marital/Relationship Status		
Single	227	27.3
In a relationship	87	10.5
Married	430	51.7
Divorced	76	9.1
Other	11	1.3
Military Service		
Any	120	14.6
None	703	85.4
Employment		
Full time	404	48.6
Part time	116	14.0
Unemployed	91	11.0
Retired	153	18.4
Students/Other	67	8.1
Income		
Less than $20,999	140	16.8
$20,000 to $39,999	158	19.0
$40,000 to $59,999	131	15.8
$60,000 to $79,999	112	13.5
$80,000 to $99,999	73	9.9
$100,000 to $149,999	105	12.6
$150,000 or more	112	13.5
**Continuous Measures**	***Mean***	**SD**
Age	44.8	16.9

## Measures

### Behavioral measures

Questions regarding COVID-related behaviors were adapted from prior surveys conducted by the CDC as well as private research groups [48–50]. Respondents were asked about their health-related behavior “recently” with respect to remaining 6 feet away from others outside of those they live with (the then-standard social distancing recommendation), avoiding groups of 10 or more persons, and wearing a mask indoors, with potential responses ranging from “Always,” “Often,” “Sometimes,” “Rarely,” to “Never.” Respondents were asked about the frequency of their activities outside the home in the past month with respect to working in an office, socializing with friends or extended family, attending religious services indoors, and going to a gym, with potential responses ranging from “Every day”, “At least weekly”, “1–3 times,” to “Never.” Finally, respondents were asked about eating at restaurants, with responses assessing whether dining was indoors or outdoors, and if it occurred regularly, infrequently, or never.

### Life satisfaction

Life satisfaction was measured using Cantril’s Ladder, a single question assessing quality of life that asks respondents to rate their current life on a 0 to 10 scale where 10 represents the best possible life for them. It is a widely used single-item measure that shows reliability and convergent validity [51–54].

### Meaning in life

Subjective meaning in life was measured using the Multidimensional Existential Meaning Scale (MEMS), a 15-item instrument that measures perceived meaning in life. Respondents answered each question on a 5-point scale, indicating their agreement with statements about meaning from 1 (Strongly Disagree) to 5 (Strongly Agree). The scale assesses the three aspects of meaning in life that are most often identified as central in the meaning literature: purpose, comprehension (i.e. coherence), and mattering [25, 30, 31]. Though relatively new, MEMS has already been used in several SWB studies (e.g., [55, 56]). MEMS is reliable and demonstrates convergent and discriminant validity. Each of its subscales helps to predict variance in other meaning in life measures, suggesting that each is crucial to the overall meaning in life construct [25].

### Analyses

Demographic predictor variables with 3 or more levels were dummy-coded prior to being entered into the model. Variables coded for white race and graduate degree-holding were removed from the model due to concerns of multicollinearity (Tolerance < 0.10; VIF > 10). Assumptions were met for univariate and multivariate normality, linearity, and normality of distributed errors were checked and met. Standard residual analyses were conducted with plots indicating that assumptions of homoscedasticity were met. Data met the assumption of independent errors (Durbin-Watson values = 2.048 and 1.99). Two hierarchical linear models (HLMs) were used to examine the potential impact of COVID-avoidant behaviors on two measures of SWB (i.e., Life Satisfaction and Meaning in Life) while accounting for the influence of demographic factors (i.e., income, age, gender, marital status, military status, and education), given documented broad influence of sociodemographic characteristics on well-being outcomes [57–59]. Accordingly, unique associations between demographic factors and SWB were tested in step 1. In step 2, COVID-avoidant behaviors were entered into the model to examine the association between such behaviors and SWB while accounting for variance attributed to demographic factors. Two-tailed significance tests (α = 0.05) were Bonferroni corrected. HLM analyses were completed using SPSS 28.

## Results

Table 2 presents the mean scores and Cronbach’s α for each subjective well-being measure. The mean Cantril’s Ladder life satisfaction score for all participants was 6.48 out of 10 (*SD* = 2.54). The mean MEMS meaning score (*n* = 771) was 3.78 out of 5 (*SD* = 0.72), with subscale means of 3.81 (*SD* = 0.83) for comprehension, 3.91 (*SD* = 0.77) for purpose, and 3.59 (*SD* = 0.77) for mattering.

**Table 2:** Subjective well-being mean scores (*n* = 831 unless specified)

Subjective Well-Being Measure	Mean Score (SD)	Cronbach’s α
Cantril’s Ladder (10-point scale)	6.48 (2.54)	-
Meaning in Life (MEMS, 5-point scale) (*n* = 771)	3.78 (0.72)	.927
Comprehension Subscale	3.81 (0.83)	.901
Purpose Subscale	3.91 (0.77)	.871
Mattering Subscale	3.59 (0.77)	.717

Table 3 presents descriptive statistics from our sample regarding COVID-avoidant behaviors during October 2020. In addition, 38.9% of respondents reported that they never ate at restaurants during the pandemic, while 11.6% reported eating only outdoors and only occasionally, 7.5% reported eating only outdoors regularly, 22.5% reported eating indoors occasionally, and 19.4% reported eating indoors regularly.

**Table 3:** COVID-avoidant behaviors among participants (*n* = 831)

**Protective behavior**	**Always**	**Often**	**Sometimes**	**Rarely**	**Never**
Social Distancing	49.0%	31.8%	12.4%	4.5%	2.4%
Avoid Crowds of 10 or More	53.1%	26.3%	13.1%	5.0%	2.5%
Wear a Mask Indoors (not at home)	65.0%	20.1%	9.1%	5.3%	3.2%
**Activities Outside the Home**	**Never**	**1–3 Times per Month**	**Once per Week**	**Every Day/** **Weekday**	
Visit Friends and/or Family	22.1%	33.2%	27.6%	17.1%	
Work at Office or Workplace	44.8%	16.9%	16.6%	26.8%	
Attend Religious Services	53.1%	12.8%	21.0%	13.1%	
Attend Gym	60.4%	11.2%	14.4%	14.1%	

To evaluate the degree to which different COVID-avoidant behaviors affected SWB when controlling for demographic characteristics (e.g., income, age, gender, marital status, military status, and education), a hierarchical linear model was computed. The first level of the model indicated that demographic covariates collectively correlated with life satisfaction scores, *F*(13, 808) = 5.93, *p* < 0.001, adjusted R^2^ = 0.072. Adding COVID-avoidant behaviors to the model significantly improved adjusted R^2^, ΔR^2^ = 0.024, ΔF(8, 800) = 2.74, *p* = 0.005, and accounted for 2.4% greater variance in life satisfaction scores. All variables together were significantly related to Cantril’s ladders scores, F(21, 800) = 4.78, p < 0.001, adjusted R^2^ = 0.088. Among COVID-avoidant behaviors, life satisfaction was significantly correlated with wearing face coverings (β = 0.087, t(800) = 2.17, *p* = 0.03), restaurant attendance (β = 0.089, t(800) = 2.03, *p* = 0.043), and attending religious services (β = -0.12, t(800) = 2.22, *p* = 0.027). Model coefficients and parameter estimates are reported in Table 4. See Supplement Table 1 for a full report of parameter estimates.

**Table 4:** Results of hierarchical regressions of Cantril’s ladder scores

Model & Dependent Variables	Adjusted *R*^2^	*b* (SE)	95% CI	β	*t*	*p*
*Model 1: Demographics*	.072					
*Model 2: Demographics* + *Behavior*	.088					
Income		.18 (.06)	[.06, .29]	.14	3.07	.002***
Gender		-.34 (.19)	[-.72, -.04]	-.07	1.77	.078
Military Service		.60 (.26)	[.10, 1.12]	.09	2.34	.019**
Age		.02 (01)	[.01, .04]	.16	3.05	.002***
Married		.31 (.10)	[.11, .52]	.13	3.04	.002***
Wear a Mask		.23 (.11)	[.02, .45]	.09	2.17	.030*
6ft Social Distancing		.12 (11)	[-.08, .33]	.05	1.17	.241
Avoid Large Groups		.01 (.11)	[-.20, .21]	.003	.07	.947
Work in Office		.05 (.09)	[-.14, .23]	.02	.49	.626
Visit Friends & Family		.19 (.11)	[-.04, .41]	.08	1.63	.103
Eat in Restaurants		.14 (.7)	[.00, .27]	.09	2.03	.043*
Go to Gym		.08 (.12)	[-.16, .32]	.04	.64	.522
Attend Religious Services		-.27 (.12)	[-.51, -.03]	-.12	-2.22	.027*

Bonferroni corrected alphas: *sig. at the .05 level **sig. at the .025 level ***sig. at the .005 level

An additional hierarchical linear regression was conducted to determine if and to what degree COVID-avoidant behaviors influenced meaning in life, as indexed by mean subjective ratings on the MEMS scale, while controlling for demographic characteristics. The first model suggested that demographic variables alone significantly affected meaning in life, F(13, 754) = 9.37, *p* < 0.001, adjusted R^2^ = 0.124. Thus, 13.9% of the variance in subjective meaning in life was accounted for by demographic variables alone. Adjusted R^2^ was significantly improved by adding COVID-related behaviors to the model, ΔR^2^ = 0.089, ΔF(8, 746) = 10.774, *p* < 0.001, and contributed 8.9% greater explanation of variance. The full model accounted for 22.8% of the variance in meaning in life ratings, F(21, 746) = 10.506, *p* < 0.001, adjusted R^2^ = 0.207. Investigation of parameter estimates indicated that after controlling for demographic variables, subjective meaning in life was significantly related to maintaining 6 feet of social distance (β = 0.11, t(800) = 2.75, *p* = 0.006) and avoiding groups of 10 or more persons (β = 0.084, t(800) = 2.09, p = 0.037). However, avoiding friends and family members due to COVID was significantly, negatively linked to meaning in life (β = -0.20, t(800) = -4.45, p < 0.001). Controlling for demographic and other COVID-avoidant behavior revealed a small significant negative correlation between meaning in life and avoiding friends and family (i.e. semipartial *r* = -0.16). Model coefficients and parameter estimates are reported in Table 5. A full report of parameter estimates is shown in Supplement Table 2.

**Table 5:** Results of hierarchical regressions of MEMS scale meaning in life scores

Model & Dependent Variables	Adjusted *R*^2^	*b* (SE)	95% CI	β	*t*	*P*
*Model 1: Demographics*	.124					
*Model 2: Demographics* + *Behavior*	.207					
Income		.04 (.02)	[.01, .07]	.12	2.59	.010**
Gender		-.05 (.05)	[-.15, .05]	-.04	-.95	.341
Military Service		.03 (.07)	[-.11, .17]	.01	.37	.713
Age		-.003 (.002)	[-.01, .00]	-.06	-1.24	.215
Married		.11 (.03)	[.05, .16]	.15	3.84	< .001***
Wear a Mask		.05 (.03)	[-.01, .11]	.07	1.81	.071
6ft Social Distancing		.08 (.03)	[.02, .13]	.11	2.75	.006**
Avoid Large Groups		.06 (.03)	[.00, .11]	.08	2.09	.037*
Work in Office		.02 (.03)	[-.03, .07]	.04	.82	.412
Visit Friends & Family		.14 (.03)	[.08, .2]	.20	4.45	< .001***
Eat in Restaurants		.03 (.02)	[-.01, .07]	.07	1.58	.116
Go to Gym		-.01 (.03)	[-.08, .6]	-.02	-.33	.741
Attend Religious Services		.02 (.03)	[-.05, .08]	.03	.54	.590

Bonferroni corrected alphas: *sig. at the .05 level **sig. at the .025 level ***sig. at the .005 level

## Discussion

### Associations with life satisfaction and meaning in life

Several altruistic anti-COVID measures were positively associated with subjective well-being. Mask wearing was significantly associated with life satisfaction, while social distancing and avoiding large gatherings were significantly associated with meaning in life. The prosocial contributions to life satisfaction of these social distancing measures may be counterbalanced to some degree by a negative impact of avoiding shallow social interactions. Meaning in life, by contrast, may not be affected by the loss of relatively shallow social interactions.

The positive association between mask wearing and life satisfaction may be a function of the prosocial nature of mask wearing, as people who feel connected with their community may be more likely to wear a mask to protect their fellow community members [60]. It is also possible that wearing a mask itself enhanced life satisfaction in the fall of 2020. It may have done so by making the wearer feel as though they were acting for the benefit of others [42–44], or by reducing anxieties regarding COVID that otherwise reduced life satisfaction among individuals who voluntarily chose not to wear a mask (e.g., [13]).

Higher levels of participation in social activities were associated, albeit non-significantly in most cases, with higher life satisfaction scores. Restaurant attendance was significantly associated with greater life satisfaction, even after adjusting for income level. Restaurant attendance may serve as a useful proxy for socialization with friends or enjoying the simple pleasures of pre-pandemic life.

Frequency of religious service attendance was negatively associated with life satisfaction scores when controlling for other variables. This was surprising, given the positive correlation found in prior studies between religious practices and life satisfaction [61, 62]. The pandemic may have reduced the number of people who regularly attend religious services, lessening the communitarian benefits of attending such services that likely play an important role in the correlation between religious practice and well-being [62]. Alternatively, persons suffering from lower life satisfaction might be more apt to risk illness to attend religious services in search of consolation.

Visiting friends and family outside of the home was associated with higher meaning in life, indicating that social and familial connections are an important foundation of meaning [28, 63]. But shallower or more casual social interactions do not appear to be a basis of meaning in life. In fact, maintaining six feet of distance from others outside the home and avoiding groups of ten persons or more were positively and significantly associated with higher meaning in life scores. This may reflect a positive relationship between engaging in altruistic behaviors and greater perceived meaning in life [44]. In addition, coupled with the positive association between visiting friends and family, it suggests that meaning in life was strongest among persons who formed small, tight-knit groups of friends and family while avoiding larger groups and maintaining social distance from strangers. In the pre-vaccination COVID era, some people formed “pods” of trusted friends and family while avoiding spending substantial time with others outside of the pod [64]. This type of social grouping, especially in difficult circumstances, may produce greater perceived meaning in life relative to other social arrangements or practices. While there is a lack of empirical evidence on the effects of such pods, some pod participants have noted the deeper social connections and feelings of closeness associated with the pods and expressed regret at their dissolution as COVID risks decreased [64].

Contrasting correlates for life satisfaction with correlates for meaning in life suggests that shallow or medium-depth social interactions and activities like dining in restaurants may be more central to life satisfaction, while deeper and narrower social interactions with family and friends are more central to meaning in life. In addition, fostering a small, tight-knit social group is likely an effective strategy for maintaining well-being during adverse world events. Such social groupings may increase perceived meaning in life and, perhaps as a result, help maintain life satisfaction [27]. Meaning in life is also a central aspect of people’s conception of a good life, independent of any effect on life satisfaction [65], and promoting it in adverse circumstances can enhance overall quality of life for an affected population. More broadly, these findings can help shed light on the foundational sources of psychological meaning or life satisfaction, a burgeoning area of research (e.g., [22, 66]). The absence of a relationship between shallow social interactions and meaning in life, for example, can help refine previous hypotheses that social interaction generally enhances perceived meaning (e.g., [31, 67]). The importance of shallow social interactions to life satisfaction may in part explain why many people to return to baseline levels of life satisfaction following the loss of a deep relationship [22].

This study’s findings, though correlational, suggest that disease prevention measures such as social distancing and mask wearing do not reduce, and may increase, SWB for adults during the early to middle stages of a pandemic. Authorities should not hesitate to encourage such measures out of concern for reduced well-being. It may also be helpful to emphasize the prosocial, charitable aspects of disease prevention measures, which could encourage people to adopt such measures or help increase SWB among those who do [42, 68].

Authorities might also use the present study as a template for measuring important aspects of subjective well-being in the midst of a pandemic. Such measures could help to gauge the psychological health of a population in real time and uncover the direction and strength of relationships between public health measures and subjective well-being. By accurately measuring the psychological effects of disease-preventive measures, authorities may be able to determine when such measures become counterproductive, or when compliance with legal mandates or non-mandatory public health guidance is likely to decline.

### Subjective well-being scores in a pandemic

The present study assessed subjective well-being in a nationally representative sample of US adults in October 2020—a critical moment in time during the pandemic. Generally, life satisfaction was relatively low compared to scores reported prior to the COVID-19 pandemic. The mean Cantril’s Ladder score of 6.48 was significantly lower than mean scores reported in comparable prior studies. It was significantly lower on a two-sample t-test than the average Cantril’s ladder scores of 6.94 (SD = 2.58, *n* = 3,000, p < 0.001) in surveys of U.S. adults conducted annually from 2017 to 2019, and the 7.03 score (SD = 2.53, *n* = 1,006, *p* < 0.001) in a survey conducted largely after March 15, 2020 [23]. This may in part reflect the tendency of respondents to report relatively higher SWB scores in live or telephone interviews and relatively lower SWB scores on questionnaires or online surveys, such as the one used in the present study [69–71]. Alternatively, it may reflect a decrease in life satisfaction or general well-being over the long course of the COVID-19 pandemic [72].

Meaning in life scores were, by contrast, relatively high compared to those reported prior to the COVID-19 pandemic. For example, on a two-sample t-test, the average MEMS score of 3.78 in the instant study was significantly higher than that reported in a pre-COVID study of 262 MTurk participants. Suh & Chong (2022), reported a mean MEMS score equivalent to 3.3 out of 5 (SD = 0.90, *p* < 0.001) [73]. Interestingly, MEMS meaning in life scores in the instant study were significantly lower on a two-sample t-test than those in a study conducted in April 2020, during the first full month of the pandemic in the United States. That study found among 575 US adults on MTurk an average MEMS score equivalent to 3.86 out of 5, significantly higher than the 3.78 score in the instant study (SD = 0.75, *p* = 0.047) [74].

These patterns may reflect a phenomenon that has begun to emerge in surveys of meaning in life: negative life events, personal struggle, and difficult situations may counterintuitively increase perceived meaning in life while also reducing subjective happiness or life satisfaction. For example, perceptions of struggle and stress may correlate with relatively higher levels of perceived meaning and lower levels of happiness [28]. Material deprivation was also correlated with higher meaning in life scores in surveys comparing average national scores between richer and poorer nations [75]. In general, people appear to engage in processes of meaning restoration following threats to their well-being [76–78]. They may do so in a variety of ways, such as interpreting a difficult situation to fit with their existing concepts of global meaning; revising their global beliefs to accommodate a new situation; achieving a sense of acceptance of a difficult new reality; and cultivating personal growth, improved relationships, better coping skills, or greater appreciation for life [79]. In studies controlling for the effects of happiness, stress and negative life events actually increased perceived meaning in life, possibly because of these meaning-making coping behaviors [28].

Reported rates of COVID-preventive behaviors were high, with a supermajority reporting frequent mask use and social distancing behaviors. This is consistent with the findings of prior studies conducted during the summer and fall of 2020 [6, 7]. Respondents reported high rates of gym and church attendance, at rates similar to those reported in non-academic surveys conducted before and during the pandemic [80–83].

### Limitations

The present study relies on self-reports of COVID-related behaviors, which may not match respondents’ actual behaviors. However, in public health studies, self-reports of COVID-related behavior were strongly and significantly correlated with objective indicators of behavior. For example, self-reported mask wearing was significantly associated with reduced COVID transmission, even when mask mandates themselves were not [8]. Likewise, self-reported social distancing measures were significantly associated with fewer steps recorded by an iPhone pedometer app activated on the phones of all study participants [84].

Respondents may overreport activities such as going to the gym because gym attendance is generally considered to be socially desirable. In addition, while our question was carefully phrased (“In the past month, how often did you go to a gym outside of your home?”) some respondents may have construed it as inquiring about how often they exercised outside of their home. Church attendance is also commonly overreported, and actual attendance may be far lower (as much as 50% lower) than reported attendance [85]. The social desirability of church attendance may motivate respondents to overreport, especially respondents who identify as religious believers [86].

One limitation of comparing our SWB scores to those reported in other studies is that, in general, comparing scores obtained in the present study to those obtained in prior studies using similar or different methods is an inexact process, as subtle differences in survey design or administration may account for some of the differences between survey scores. Further surveys of subjective well-being during various points of the pandemic and the post-pandemic era would help to confirm the effects discussed here.

The present study did not assess respondents’ Big-5 personality traits (e.g., [87]). Accordingly, it cannot rule out the possibility that personality traits drive both COVID-related behavior and SWB levels, confounding the relationship between them. This limitation regarding the potentially confounding nature of personality is a common one in studies of behavior and SWB [28, 42, 43, 63, 88]. In addition, individuals with high levels of life satisfaction may value their lives more highly and be more likely to take preventative health measures such as wearing a mask. While we posit that mask wearing is more likely correlated with prosociality or directly improves life satisfaction by alleviating anxiety, we cannot rule out this possibility.

An additional limitation of the present study was that it did not determine respondents’ political affiliations. COVID-avoidant behaviors likely differed based on the political affiliations of individuals even prior to vaccine roll-outs. For example, Gollwitzer et al. (2020), reported that US counties that voted for Donald Trump over Hillary Clinton in 2016 exhibited 14% less physical distancing as measured by smartphone geolocation data between March and May 2020 [89]. The present study cannot rule out that political differences might drive both divergent COVID-related behavior and differences in SWB scores. Evidence is mixed on the relationship of partisanship to SWB [90, 91]. Some studies have indicated that self-identified conservatives generally report higher SWB than self-identified liberals [92], while more recent studies have found no significant difference [93]. It is possible that conservative respondents were more likely to report higher SWB, and this effect may account for the positive associations reported for restaurant attendance and visiting friends and family. However, the theory that conservatism is driving both fewer COVID precautions and higher life satisfaction scores is not consistent with our data showing that mask wearing is positively related to life satisfaction scores, or that church attendance [94] is negatively related to such scores. Likewise, a theory that conservatism explains both fewer COVID precautions and higher meaning in life is not consistent with the correlation between social distancing practices and higher meaning in life scores.

## Conclusion

Although the COVID pandemic is constantly changing, as are people’s behavioral responses to it, major world events can provide opportunities for researchers to examine relationships between subjective well-being and behavior in unique contexts. The present study examines these relationships in the unique environment of the United States in October 2020. It finds significant positive relationships between COVID-preventive behaviors and subjective well-being. This study’s findings support the conclusion that disease prevention measures such as social distancing and mask wearing do not reduce, and may enhance, subjective well-being during a pandemic.

Further, by comprehensively assessing subjective well-being in the midst of a pandemic, the present study can help shed light on how populations respond psychologically to adverse, nationwide events. The study indicates that shallower social activities are likely to be more central to life satisfaction, while narrower, deeper social interactions are likely to be more important to perceived meaning in life.

### Supplementary Information

**Additional file 1:** **Supplement Table 1.** Results of Full Hierarchical Regressions of Cantril’s Ladder Scores. **Supplement Table 2.** Results of Full Hierarchical Regressions of MEMS Scale Meaning in Life Scores.

## Data Availability

The dataset supporting the conclusions of this article is available in the figshare repository, 10.6084/m9.figshare.21514056.

## Abbreviations

MEMS: Multidimensional existential meaning scale
SWB: Subjective well-being
