# Supplementary material for: Disease-preventive behaviors and subjective well-being in the COVID-19 pandemic
Source: BMC Psychol. 2023 Sep 25;11:288. doi: 10.1186/s40359-023-01316-x (PMC10521491; doi:10.1186/s40359-023-01316-x)
Supplement: Supplementary file 1 — Additional file 1: Supplement Table 1. Results of Full Hierarchical Regressions of Cantril’s Ladder Scores. Supplement Table 2. Results of Full Hierarchical Regressions of MEMS Scale Meaning in Life Scores. [file 40359_2023_1316_MOESM1_ESM.pdf]

Social Indicators Research

Disease-preventive behaviors and subjective well-being in the COVID-19 pandemic

Matthew Tokson[a]*, Hadley Rahrig[b], Jeffrey D. Green[c]

[a]*S.J. Quinney College of Law, University of Utah, Salt Lake City, Utah, USA, ORCID: 0000-0002-3192-173X;* [b] *Center for Healthy Minds, University of Wisconsin-Madison, Madison, Wisconsin, USA, ORCID: 0000-0003-0175-8262;* [c] *Psychology Department, Virginia Commonwealth University, Richmond, Virginia, USA*

Supplement Table 1

Results of Full Hierarchical Regressions of Cantril's Ladder Scores

Model & Dependent Variables	Adjusted R^2	*b* (SE)	95% CI	β	*t*	*p*
Model 1: Demographics	.072					
Income		.18 (.06)	[.07, .29]	.15	3.19	.001***
Gender		-.38 (.19)	[-.76, -.00]	-.08	-1.973	.049*
Military Service		.53 (.27)	[.03, 1.03]	.07	2.06	.040*
Age		.02 (.01)	[.01, .04]	.16	3.50	<.001***
Married		.34 (.10)	[.14, .54]	.14	3.33	<.001***
Education – HS		.16 (.30)	[-.43, .75]	.03	.52	.601
Education - As. Deg. / Some Col.		-.30 (.28)	[-.86, .25]	-.05	-1.08	.282
Education – Col. Deg.		-.23 (.26)	[-.74, .28]	-.04	-.89	.374
Race – AIAN		-.08 (.54)	[-1.14, .98]	-.01	-.15	.881
Race – Asian		.12 (.40)	[-.65, .90]	.01	.31	.754
Race – Black or AA		.55 (.33)	[-.10, 1.20]	.07	1.67	.095
Race – His. or Lat.		.41 (.29)	[-.16, .99]	.06	1.40	.161
Race – Other		-.74 (.67)	[-2.07, .58]	-.04	-1.1	.272
Model 2: Demographics + Behavior	.088					
Income		.18 (.06)	[.06, .29]	.14	3.07	.002***
Gender		-.34 (.19)	[-.72, -.04]	-.07	1.77	.078
Military Service		.60 (.26)	[.10, 1.12]	.09	2.34	.019**
Age		.02 (01)	[.01, .04]	.16	3.05	.002***
Married		.31 (.10)	[.11, .52]	.13	3.04	.002***
Education – HS		.33 (.30)	[-.27, .92]	.06	1.07	.286
Education - As. Deg. / Some Col.		-.22 (.29)	[.79, .34]	-.04	-.78	.434
Education – Col. Deg.		-.13 (.26)	[-.64, .38]	-.02	-.50	.617
Race – AIAN		-.05 (.54)	[-1.12, 1.01]	-.003	-.01	.921
Race – Asian		.16 (.40)	[-.62, .95]	.02	.41	.685
Race – Black or AA		.66 (.33)	[.01, 1.31]	.09	1.98	.048*
Race – His. or Lat.		.44 (.30)	[-.15, 1.02]	.07	1.47	.143
Race – Other		-.59 (.68)	[-1.92, .74]	-.03	-.87	.387
Wear a Mask		.23 (.11)	[.02, .45]	.09	2.17	.030*
6ft Social Distancing		.12 (11)	[-.08, .33]	.05	1.17	.241
Avoid Large Groups		.01 (.11)	[-.20, .21]	.003	.07	.947
Work in Office		.05 (.09)	[-.14, .23]	.02	.49	.626
Visit Friends & Family		.19 (.11)	[-.04, .41]	.08	1.63	.103
Eat in Restaurants		.14 (.7)	[.00, .27]	.09	2.03	.043*
Go to Gym		.08 (.12)	[-.16, .32]	.04	.64	.522
Attend Religious Services		-.27 (.12)	[-.51, -.03]	-.12	-2.22	.027*

Bonferroni corrected alphas: *sig. at the .05 level **sig. at the .025 level ***sig. at the .005 level

Supplement Table 2

Results of Full Hierarchical Regressions of MEMS Scale Meaning in Life Scores

Model & Dependent Variables	Adjusted R^2	b (SE)	95% CI	β	t	p
Model 1: Demographics	.124					
Income		.05(.02)	[.02, .08]	.14	3.00	.003***
Gender		-.08 (.05)	[-.18, .03]	-.05	-1.4	.164
Military Service		-.06 (.07)	[-.20, .08]	-.03	-.82	.415
Age		-.01 (.002)	[-.01, .00]	-.11	-2.42	.016**
Married		.13 (.03)	[.07, .18]	.18	4.37	<.001***
Education – HS		-.06 (.09)	[-.23, .11]	-.04	-.72	.475
Education – As. Deg. / Some Col.		-.16 (.08)	[-.32, .00]	-1.0	-2.00	.050*
Education – Col. Deg.		-.09 (.03)	[-.24, .05]	-.06	-1.28	.212
Race - AIAN		.018 (.15)	[-.28, .32]	.02	.454	.905
Race – Asian		.06 (.11)	[-22, .21]	-.002	-.05	.962
Race – Black or AA		-.07 (.09)	[-.25, .11]	-.034	-.77	.442
Race – His. or Lat.		-.06 (.08)	[-.21, .10]	-.03	-.69	.491
Race – Other		-.30 (.18)	[-.67, .06]	-.06	-1.64	.101
Model 2: Demographics + Behavior	.207					
Income		.04 (.02)	[.01, .07]	.12	2.59	.010**
Gender		-.05 (.05)	[-.15, .05]	-.04	-.95	.341
Military Service		.03 (.07)	[-.11, .17]	.01	.37	.713
Age		-.003 (.002)	[-.01, .002]	-.06	-1.24	.215
Married		.11 (.03)	[.05, .16]	.15	3.84	<.001***
Education – HS		.06 (.08)	[-.10, .22]	.04	.72	.473
Education – As. Deg. / Some Col.		-.06 (.08)	[-.22, .09]	-.04	-.79	.432
Education – Col. Deg.		.004 (.07)	[-.14, .15]	.002	.05	.958
Race – AIAN		.07 (.15)	[-.22, .35]	-.02	.45	.650
Race – Asian		.07 (.11)	[-.17, .28]	.02	.61	.542
Race – Black or AA		-.04 (.09)	[-.22, .10]	-.02	-.45	.652
Race – His. or Lat.		-.06 (.08)	[-.22, .10]	-.03	-.69	.491
Race – Other		-.30 (.19)	[-.67, .06]	-.06	-1.64	.101
Wear a Mask		.05 (.03)	[-.01, .11]	.07	1.81	.071
6ft Social Distancing		.08 (.03)	[.02, .13]	.11	2.75	.006**
Avoid Large Groups		.059 (.028)	[.003, .11]	.08	2.09	.037*
Work in Office		.02 (.03)	[-.03, .07]	.04	.82	.412
Visit Friends & Family		.14 (.03)	[.08, .2]	.20	4.45	<.001***
Eat in Restaurants		.03 (.02)	[-.01, .07]	.07	1.58	.116
Go to Gym		-.01 (.03)	[-.08, .6]	-.02	-.33	.741
Attend Religious Services		.02 (.03)	[-.05, .08]	.03	.54	.590

Bonferroni corrected alphas: *sig. at the .05 level **sig. at the .025 level ***sig. at the .005 level
